# Occurrence and Spread of the Invasive Asian Bush Mosquito *Aedes japonicus japonicus* (Diptera: Culicidae) in West and North Germany since Detection in 2012 and 2013, Respectively

**DOI:** 10.1371/journal.pone.0167948

**Published:** 2016-12-09

**Authors:** Helge Kampen, Cornelius Kuhlisch, Andreas Fröhlich, Dorothee E. Scheuch, Doreen Walther

**Affiliations:** 1 Friedrich-Loeffler-Institut, Federal Research Institute for Animal Health, Greifswald–Insel Riems, Germany; 2 Leibniz Centre for Agricultural Landscape Research, Müncheberg, Germany; New Mexico State University, UNITED STATES

## Abstract

The invasive Asian bush mosquito *Aedes japonicus japonicus* was first recognised as established in Germany in 2008. In addition to the first known and quickly expanding population in the southwestern part of the country, three separate populations were discovered in West, North and southeastern Germany in 2012, 2013 and 2015, respectively, by means of the ‘Mueckenatlas’, a German instrument of passive mosquito surveillance. Since the first findings of mosquito specimens in West and North Germany, these regions were checked annually for continuing colonisation and spread of the species. Both affected areas were covered by a virtual 10x10km^2^ grid pattern in the cells of which cemeteries were screened for immature stages of the mosquito. The cells were considered populated as soon as larvae or pupae were detected, whereas they were classified as negative when no mosquito stages were found in the cemeteries of at least three different towns or villages. Presence was also recorded when *Ae*. *j*. *japonicus* adults were submitted to the ‘Mueckenatlas’ from the respective cell or when there was evidence of local occurrence in localities other than cemeteries. Based on this approach, a significant expansion of the populated area was documented in West Germany since the first detection of *Ae*. *j*. *japonicus* in 2012 (increase in positive grid cells by more than 400%), while the North German population appears not to be expanding so far (reduction of positive grid cells by ca. 30% since 2013). As *Ae*. *j*. *japonicus* finds suitable climatic and ecological conditions in Germany, the differential expansion of the two populations might be attributed to the West German population being older and thus more firmly established than the closely related but younger North German population that might still be in its founder phase. However, geographic spread of all German populations in the future is anticipated. Continuous surveillance is recommended, as *Ae*. *j*. *japonicus* is a competent vector of several pathogens in the laboratory.

## Introduction

As a result of globalisation and the worldwide trade with used tyres, lucky bamboo and water-holding machinery, *Aedes* mosquitoes are regularly transported around the world and introduced into non-endemic areas [1]. The Asian bush mosquito *Aedes* (*Hulecoeteomyia*) *japonicus japonicus* is one of the top-ranked invasive mosquito species of the world [2]. Originating from East Asia (Korea, Japan, Taiwan, southern China, southeastern Russia), where winters can be extremely cold [3], *Ae*. *j*. *japonicus* is well adapted to climatic conditions in certain parts of North America and in Central Europe. It showed up in the United States in the 1990s where it is now widely distributed in 33 states including Hawaii [4]. In 2001, it was detected farther north in Canadian Quebec and Ontario [5, 6], in 2013 in Newfoundland [7] and in 2014 in Vancouver, British Columbia [8]. In Europe, *Ae*. *j*. *japonicus* was first established in Belgium in 2002, followed by Switzerland, Germany, Austria, Slovenia, The Netherlands, Croatia, Hungary and France between 2008 and 2013 [4]. At present, there are seven apparently separate populations in Europe with the Belgian population being the only one with evidence for its mode of introduction as it is restricted to the premises of two used tyre-trade companies [9]. By contrast, little information exists on where the other European populations came from, how and where they entered Europe and how they dispersed within Europe. Molecular population analyses showed two microsatellite genetic signatures in Europe suggesting that at least two independent introduction events took place [10, 11].

**Fig 1:**
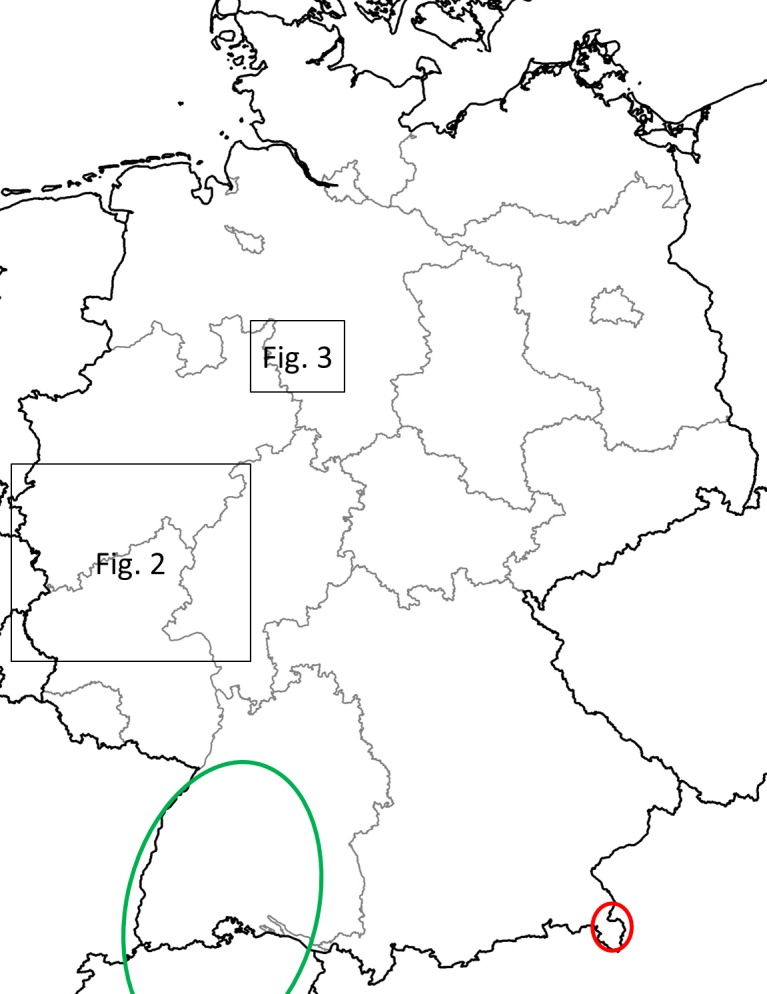
Map showing the geographic locations of the four known *Ae*. *j*. *japonicus* populations in Germany (population encircled in green was neither detected nor studied by the authors; for population encircled in red see [15]). Insets mark the two populations/areas surveyed.

In Germany, four distinct *Ae*. *j*. *japonicus* populations have been detected, one in 2008 in the southwestern part of the country, one in 2012 in the central western region, one in 2013 in a more northern area, and one in 2015 in the Southeast [12–15] (Fig 1), with distances of at least 190km between the closest known boundaries of these in 2013. According to genetic analyses by Zielke et al. [11, 15], the northern and the southeastern German populations are probably offshoots of the West German and the Austrian/Slovenian populations, respectively, while the Southwest German population, extending cross-border into Switzerland and France [16, 17], has a different genetic makeup [10, 11, 15].

Although the species naturally breeds in tree holes and rock pools, it accepts a wide variety of artificial water containers such as rain water casks, jars, flower vases, pots and dishes [18]. This marked lack of specificity in choice of breeding site and environment provides *Ae*. *j*. *japonicus* with excellent developmental opportunities and is the reason why the species can also be found in cemeteries, either in urban, suburban or near natural settings (e.g. forests).

Cemeteries are opportune facilities from both the mosquito’s and the investigator’s point of view [19]: they usually offer not only large quantities of breeding sites but also appropriate habitats for adult mosquitoes such as bushes and trees that provide shade and shelter. Moreover, food sources for adult mosquitoes are present in the form of flowering plants and blood hosts (human visitors, birds and small mammals). For the collector, cemeteries are easily accessible (in contrast to private premises), and a large number of potential breeding sites can be checked for larvae and pupae within a limited period of time.

*Aedes j*. *japonicus* is considered a potential vector of disease agents although its vector competencies have mainly been demonstrated in the laboratory. Experimentally, it was able to transmit Japanese encephalitis, West Nile, dengue, chikungunya, Rift Valley fever and Getah viruses [20–24], while in the field, it was found infected with Japanese encephalitis, West Nile and La Crosse viruses [25–27]. In addition to being a potential vector, the species is suspected to replace indigenous mosquito species once established in a new area [28, 29].

The West and North German *Ae*. *j*. *japonicus* populations dealt with in this study were discovered in 2012 and 2013, respectively, by means of the ‘Mueckenatlas’, a citizen science project addressing the spatiotemporal mapping of mosquito species in Germany [30]. The purpose of the present study was to track the spread of these two populations since their detection, using regular field collections of mosquito developmental stages.

## Materials and Methods

### Mosquito collection

The findings presented are based on two surveillance approaches, a passive and an active one. In the scope of the passive surveillance instrument ‘Mueckenatlas’, citizens are requested to collect adult mosquitoes in their private surroundings, kill them by freezing overnight and send them by post to the research institutions involved [30]. Upon arrival, they are identified to species morphologically or, if necessary (i.e. in the case of damaged and incomplete specimens or cryptic species), genetically. The ‘Mueckenatlas’ was launched in April 2012, and to date mosquitoes from all over Germany have been submitted, including numerous *Ae*. *j*. *japonicus* specimens from their four German distribution areas.

The active surveillance approach, initially a reaction to the submission of *Ae*. *j*. *japonicus* from West and North Germany, includes on-site monitoring by visiting areas suspected of being colonised and then searching for mosquito larvae and pupae. After reception of the first *Ae*. *j*. *japonicus* specimens from West and North Germany, the immediate surroundings of their collection sites (usually private gardens) were inspected for water containers harbouring immature stages of the species, followed by the closest cemeteries. When it became clear that *Ae*. *j*. *japonicus* had become established, further surveillance was based on a grid pattern of 10x10km^2^ cells virtually laid over the affected region. Generally, up to three cemeteries per cell were checked for the presence of *Ae*. *j*. *japonicus* developmental stages by scrutinizing flower vases, supply wells, bowls and other artificial water-holding containers. If larvae were found, all cells surrounding the positive cell (sometimes more) were subjected to the same procedure. If no larvae could be detected in three cemeteries of different villages or towns in the same grid cell, the cell was considered negative. A positive cell was also recorded for any case of *Ae*. *j*. *japonicus* found outside of a cemetery, i.e. by ‘Mueckenatlas’ sample submissions or arbitrary larval findings in gardens or wooded areas.

The inspected cemeteries, which were selected at random by exploring villages, towns or city districts in the desired grid cell, varied in size, number and type of potential breeding sites available, and density of vegetation. As these fixed parameters rendered the various sampled cemeteries incomparable, it was not considered appropriate to examine the same number of water containers in each cemetery. Instead one hour at most was spent in a cemetery, with every flower vase, dish, bowl and other container with water checked in smaller cemeteries and at least 80 water containers checked in larger ones. The selection of the inspected containers was also arbitrary but focused on vases under beech trees (*Fagus* spec.) and in other shaded and vegetated areas if present. Also, containers were checked in different sectors of large cemeteries. The search was stopped immediately when larvae of *Ae*. *j*. *japonicus* were unambiguously identified by specific morphological and behavioural characteristics [13]. This identification in the field was later confirmed in the laboratory genetically by CO1 barcoding [31, 32] of alcohol-fixed larvae from every site considered positive. In cases of uncertainty (e.g. early stage larvae), individuals suspected of being *Ae*. *j*. *japonicus* were transferred to beakers together with some water they were developing in, and kept until adult emergence. Adults were then identified to species morphologically [33]. Should immediate species identification have been impossible in the field, the situation was equalised with the non-finding of *Ae*. *j*. *japonicus*, and the search was continued as described.

After the first detections of *Ae*. *j*. *japonicus* in West and North Germany, the two affected areas (Fig 1) were repeatedly surveyed, if possible twice a year, once in May and once in August, based on the observation in areas of similar climatic conditions in the United States that there are two larval population peaks (spring and mid- to late summer) during the breeding season [34]. Population densities were not determined but efforts necessary to find *Ae*. *j*. *japonicus* larvae can be conveyed by the numbers of cemeteries that had to be examined.

### Statistics

The probability of *Ae*. *j*. *japonicus* being present and detected of in the cemeteries can be described as follows: supposing that the larvae are evenly spread over an infinite number of potential breeding sites, their detection has a probability of 99% (95%) as long as at least 5.6% (3.7%) of the examined sites are populated (binomial model). Given the documented expertise and experienced collecting approach of the persons looking for the mosquitoes and the genetic identification techniques applied, the specificity of correct species determination can be considered 100%. False positive results were, therefore, excluded.

To determine the spatiotemporal expansion of the area colonised by *Ae*. *j*. *japonicus*, the sign test was applied. As all grid cells were of equal size, only the ratio of positive and negative grid cells within a defined area needed consideration.

To check for the difference in investigation effort between the populated areas and the years of data collection, the chi-square test was applied to counts of cemetery visits per grid cell necessary to find *Ae*. *j*. *japonicus* aquatic stages in relation to the maximum possible number of visits (which was preset at three).

For both the sign test and the chi-square test, significance was set at p<0.05.

All analyses were performed in R [35].

## Results

### West German population

**Fig 2:**
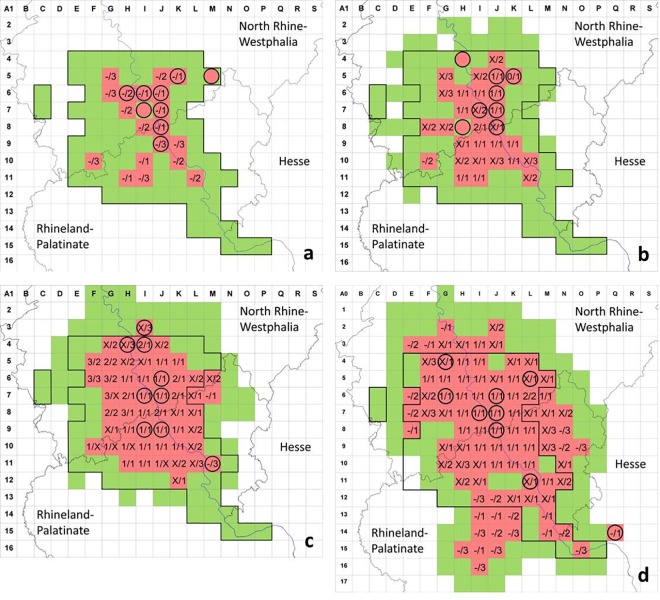
Area of West Germany in the federal states of North Rhine-Westphalia, Rhineland-Palatinate and Hesse checked for *Ae*. *j*. *japonicus* in 2012 (a), 2013 (b), 2014 (c) and 2015 (d). Red squares: grid cells positive, green squares: grid cells negative by cemetery inspection, circles: grid cells with *Ae*. *j*. *japonicus* submissions to the ‘Mueckenatlas’; figures in the grid cells denote findings in May/August of the respective year with ‘1’, ‘2’, ‘3’ representing the number of cemeteries inspected until *Ae*. *j*. *japonicus* was found, ‘X’ representing three negative cemeteries in case only one of the two collection seasons was positive, and ‘-‘ meaning not sampled. The area framed in bold marks the reference area used for statistical analysis.

The first *Ae*. *j*. *japonicus* specimens from West Germany were submitted to the ‘Mueckenatlas’ in July 2012. Based on a 10x10km^2^ grid pattern, the area affected was checked for developmental mosquito stages in August 2012, May and August 2013, May and August 2014, and May and August 2015 (S1 Table). The findings differed between the two inspections per year and have been summed for each year in Fig 2.

According to detections of specimens in the field, *Ae*. *j*. *japonicus* spread considerably from 2012 to 2015 (Fig 2). Numbers of grid cells with larval findings increased from 21 in 2012 to 31 in 2013 (plus 47.6%), 52 in 2014 (plus 67.7%) and 89 in 2015 (plus 71.2%), totalling 424% from 2012 to 2015 (Table 1). Although population densities were not determined, larvae could generally be found with less effort in the centre of the populated area than in peripheral areas (Fig 2A–2D), where they were often detected only after extensive search and in limited numbers (sometimes only two specimens per cemetery).

**Table 1:** Number of grids cells positive for *Ae*. *j*. *japonicus* (number of ‘Mueckenatlas’ collection sites:number of submitted specimens are given in parentheses).

	2012		2013			2014			2015		
	August	Total*	May	August	Total*	May	August	Total*	May	August	Total*
**West Germany**											
‘Mueckenatlas’	7 (10:14)	21	---	8 (11:27)	31	---	9 (14:56)	52	---	8 (13:16)	89
field sampling	19		13	29		33	48		32	89	
**North Germany**											
‘Mueckenatlas’	---	---	---	1	12	---	1	10	---	2	8
field sampling	---	---	11	9		6	10		---	7	

*As the same grid cells may have been positive in both collection periods per year and/or by both collection approaches, totals are less than sums.

With few exceptions, ‘Mueckenatlas’ submissions could be verified by mosquito demonstrations in the field. In both 2012 and 2013, however, two cells (M5 and I7 in 2012, H4 and H8 in 2013) were positive by ‘Mueckenatlas’ submission whereas no *Ae*. *j*. *japonicus* individuals could be found in cemeteries of the respective cells (Fig 2A and 2B). Cell M5, from which an *Ae*. *j*. *japonicus* specimen had been submitted to the ‘Mueckenatlas’ in 2012, remained negative until 2014 (Fig 2C), and only in 2015 were larvae demonstrated in this cell (Fig 2D), although in a cemetery of a town different from where the ‘Mueckenatlas’ submission originated.

Generally, the numbers of grid cells rated positive in August were higher than those in May (Table 1) although not all cells positive in May were necessarily positive in August. For example in May 2014, five cells in the periphery of the populated area (G7, F10, G10, H10, J11) were negative in August (Fig 2C).

Similarly, most cells positive in one year were confirmed positive in the next. As an exception, *Ae*. *j*. *japonicus* larvae could not be detected in 2015 in five cells that had been positive in 2014 (J4, G8, F10, J11, K11; Fig 2C and 2D). Four of these (J4, G8, J11, K11) and some other negative cells (F8, M10, L13, L14) were completely or predominantly surrounded by positive cells in 2015 (Fig 2D), causing a somewhat patchy picture of colonisation.

Cell Q14 was only checked in early September 2015 due to three submissions of *Ae*. *j*. *japonicus* to the ‘Mueckenatlas’ in August. While the species was eventually found in two cemeteries in that cell, the surrounding cells could not be sampled in the expiring mosquito season. The negative cells east of cells N14 and O15 therefore convey the visual impression that positive cell Q14 is isolated from the West German population (Fig 2D).

The only grid cell with *Ae*. *j*. *japonicus* findings beyond a cemetery during the field surveys was cell I3 in August 2014, where larvae were detected in numerous tree holes in a beech forest. Shortly before, several adult specimens collected in this forest had been submitted to the ‘Mueckenatlas’. After the breeding sites were identified, a cemetery about 2km away in direct line was also shown to be colonised in August 2014 whereas another cemetery in the same cell, 2.4km away, seemed to be free of *Ae*. *j*. *japonicus*.

The annual increase in grid cells positive for *Ae*. *j*. *japonicus*, as initially based on subjective observations and interpreted as a spreading mosquito population, is supported statistically. To check the hypothesis of a geographic spread of the mosquito population within a defined area, the area examined in 2012 was annually evaluated (Fig 2A). Within this area (framed in bold in Fig 2A–2D), all cells were assessed regarding their observed status compared to the previous year. The potential status of the two cells not sampled within the frame in 2015 (E10, E11), either positive or negative, had no effect on the outcome of the statistical analysis. The hypothesis that the ratio of positive and negative squares remains stable from one year to the following must be rejected according to the sign test (p> 0.05), whereby multiple testing (year-to-year comparison) is considered. From 2012 to 2013, for example, 12 shifts from negative to positive were registered, but only one shift from positive to negative (p = 0.0017). Similarly, 18 shifts from negative to positive versus one shift from positive to negative were observed for the period 2013−2014 (p<0.0001), and 16 shifts from negative to positive versus 5 shifts from positive to negative for the period 2014−2015 (p = 0.0133).

In the West German population, 226 cemeteries had to be visited in 82 cells of the reference area in August 2012 to find *Ae*. *j*. *japonicus* larvae, according to the preset criteria, averaging 2.76 cemeteries per cell. Only 140 cemeteries were inspected in 80 cells in August 2015, averaging 1.75 visits per cell. Thus, the examination effort was considerably less in 2015 compared to 2012 (chi-square test: Χ^2^ = 8.2428, df = 1, p = 0.00409). The effort increased when examining less populated peripheral areas.

### North German population

**Fig 3:**
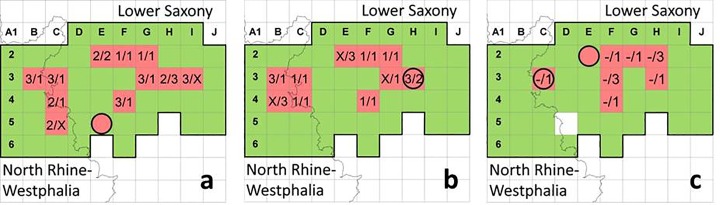
Area of North Germany in the federal states of North Rhine-Westphalia and Lower Saxony checked for *Ae*. *j*. *japonicus* in 2013 (a), 2014 (b) and 2015 (c). Red squares: grid cells positive, green squares: grid cells negative by cemetery inspection, circles: grid cells with *Ae*. *j*. *japonicus* submissions to the ‘Mueckenatlas’; figures in the grid cells denote findings in May/August with ‘1’, ‘2’, ‘3’ representing the number of cemeteries inspected until *Ae*. *j*. *japonicus* was found, ‘X’ representing three negative cemeteries in case only one of the two collection seasons was positive, and ‘-‘ meaning not sampled. The area framed in bold marks the reference area used for statistical analysis.

A specimen of *Ae*. *j*. *japonicus* from North Germany was submitted to the ‘Mueckenatlas’ for the first time in late summer 2012, but the collection area could be visited only in May 2013. As for West Germany, the annual extent of geographic spread was assessed based on cemetery inspection in a 10x10km^2^ grid pattern. Survey data exist from May and August 2013 and May and August 2014, while only the August field study could be carried out in 2015 (S2 Table). For 2013 and 2014, the data of the two annual surveys were again summed for Fig 3.

The situation for the North German *Ae*. *j*. *japonicus* population was different from that in West Germany in that the area colonised (Fig 3A–3C) decreased in the three years of observation. A reduction from 12 positive grid cells in 2013 to 10 in 2014 (reduction of 16.7%) and 8 in 2015 (reduction of 20%) was registered, giving a total reduction of one third from 2013 to 2015 (Table 1). Over the years, only five cells (F2, G2, C3, H3, F4) were consistently positive, and in none of the years did the cells display a spatially non-disrupted population. Despite this, *Ae*. *j*. *japonicus* larvae were found in two cells in 2015 (H2, F3) where no specimens had been detected previously.

In 2013 and 2014 (Fig 3A and 3B), one submission each to the ‘Mueckenatlas’ was registered, while in 2015 (Fig 3C) two submissions were received. The submission in 2013 and one of the submissions in 2015 did not correlate with positive cemetery inspections in the respective cells.

As in West Germany, cells usually remained positive in August when they had been positive in May of the same year. Cells I3 and C5 were exceptions in 2013, and 2013 was the only year with more cells positive in May than in August (Table 1).

Except for 2012 in West Germany, the numbers of cemeteries per cell that had to be checked to find *Ae*. *j*. *japonicus* were higher in North Germany than in West Germany, suggesting that the population density was generally lower (Fig 3A–3C). In North Germany, a minimum of 2.69 cemeteries had to be visited per cell in August 2013 until *Ae*. *j*. *japonicus* was found. In that collection period, a total of 129 cemeteries were inspected in 48 cells which increased to 137 cemeteries in 49 cells in August 2015 (2.80 visits per cell). A statistical difference as to the annual average examination effort per cell was not found (chi-square test: Χ^2^ = 0.0028, df = 1, p = 0.9577). Both measurements (i.e., August 2013 and August 2015) can therefore be considered jointly and result in an average collection effort of 2.74 cemetery visits per cell. This value corresponds to the efforts in the West German study area in August 2012 (chi-square test: Χ^2^ = 0.0199, df = 2, p = 0.099).

Despite the decline of two positive grid cells per year in the North German *Ae*. *j*. *japonicus* distribution area, the hypothesis that the ratio of negative and positive squares are comparable over time, corresponding to a more or less stable population area, cannot be rejected (p>0.1).

## Discussion

The emergence and spread of the invasive Asian bush mosquito *Ae*. *j*. *japonicus* in North America and Europe are attributed to the broad ecological tolerance and adaptability of this mosquito species [36]. Particularly, the resistance of its eggs to low temperatures, an extended season of activity from early spring to late autumn and the low grade of specialisation in the choice of breeding sites, with the immature stages tolerating high organic concentrations, support the survival and establishment of the species in non-native areas [9].

Not only are the eggs of *Ae*. *j*. *japonicus* dispersed by continental and intercontinental transport leading to subsequent success in establishing new populations at some of their destinations, but populations may also quickly expand once firmly established. The factors determining an increase in population densities and a subsequent geographic spread of the population are not clear, and there is evidence that populations remain more or less static in terms of area coverage over many years, e.g. in Belgium [9]. Observations from the United States suggest that *Ae*. *j*. *japonicus* might need one to three years for breeding site numbers and population densities, and thus detection frequencies, to significantly increase [37].

We tried to assess the continuing presence and the rate of geographic spread of *Ae*. *j*. *japonicus* populations by checking cemeteries in a grid cell pattern. Although this approach lacks standardisation, and thus comparability, it has successfully been applied in various studies targeting *Aedes* species and enables a good overview of their spatial distribution, e.g. [16, 38]. Accordingly, *Ae*. *j*. *japonicus* showed a significant spread of a population in West Germany but a more or less static population in North Germany between 2012 and 2015, and 2013 and 2015, respectively.

Although no specific data on population densities were collected during the years of observation, generally more cemeteries needed to be checked to find *Ae*. *j*. *japonicus* specimens and the numbers of specimens encountered were much lower in peripheral grid cells than in central grid cells in West Germany. The same situation applies to the comparison of the North and the West German populations in that efforts to find larvae were comparable between most grid cells in the North German population and cells in the periphery of the West German population. Thus, based on the finding that the genetic makeup of the North German population suggests a close relationship with the West German population, Zielke et al. [11] concluded that the North German population is an offshoot of the West German one, probably as a consequence of passive vehicle transport of founder individuals along a connecting motorway. Therefore, the North German population is likely to be younger than the West German one and probably not as firmly established.

The assumption that the West and North German populations are of different ages is also supported by the area coverage, as expressed by the number of positive grid cells. This number was generally lower in May than in August, probably due to low population densities at the beginning of the season. Probably, low abundance and patchy occurrence, rather than absence, were the reasons for cells without *Ae*. *j*. *japonicus* surrounded by cells with findings.

*Aedes j*. *japonicus* could not be found in the cemetery of the town of the 2012 ‘Mueckenatlas’ submission from grid cell M5 in West Germany. Specimens were only found in a cemetery of another town of that cell in 2015. It is possible that *Ae*. *j*. *japonicus* has occurred in that cell since 2012 but in a population density below the detection limit. Despite the expansion of the West German populated area, grid cell M5 remained in its periphery, where abundances must be assumed to have been very low even in late August, until 2015. Alternatively, *Ae*. *j*. *japonicus* might not have been discovered in that cell because it did not colonise cemeteries. In grid cell I3 in West Germany, for example, *Ae*. *j*. *japonicus* was first detected as adults in a beech forest in 2014 and sent for identification. An on-site inspection showed the species to be widely distributed in tree holes in that forest. In the closest cemetery, however, it could not be found, and only a few specimens were detected in the second closest cemetery.

*Aedes j*. *japonicus* has the competitive advantage over several indigenous mosquito species with similar ecological niches by being active from very early until very late in the season [36]. It can therefore quickly develop high abundances in the centre of established populations and might outcompete indigenous mosquito species [34]. In central grid cells of the West German population, masses of larvae were observed in water basins of several cemeteries in early April while few or no specimens of other species could be found. The water temperatures at that time, which were generally below 10°C and as low as 4°C (unpublished data), agree with the onset of larval development at 4.5–5°C as measured in southern New Hampshire, USA, by Burger & Davis [34].

In summary, *Ae*. *j*. *japonicus* as an intruder does not appear to have competitive disadvantages as opposed to indigenous mosquito species. It is highly adaptable to the German climate and tends to expand as soon as certain population densities are reached. This is the case in the West German population, probably as well as in the southwestern German population, but not yet in the North German population. The results indicate that the speed of active spread is rapid once a population is firmly established. The West German population is, therefore, predicted to breach the border to Belgium in the west and to merge with the Southeast German population in the near future, possibly already in 2016. This may lead to a highly mixed ‘superpopulation’ with broad genetic diversity and, thus, an even greater adaptability. Although *Ae*. *j*. *japonicus* can no longer be eradicated from Germany and must now be considered a permanent component of the country’s mosquito fauna, further monitoring might produce valuable information on the establishment and spatiotemporal expansion of an invasive mosquito species as well as a potential vector of disease agents.

## Supporting Information

S1 Table Location, grid cell assignment and monitoring results of the sites checked for *Ae*. *j*. *japonicus* in West Germany. (XLSX)

S2 Table Location, grid cell assignment and monitoring results of the sites checked for *Ae*. *j*. *japonicus* in North Germany. (XLSX)
